# Excitatory Effects of Calcitonin Gene-Related Peptide (CGRP) on Superficial Sp5C Neurons in Mouse Medullary Slices

**DOI:** 10.3390/ijms22073794

**Published:** 2021-04-06

**Authors:** Fang Zheng, Barbara E. Nixdorf-Bergweiler, Johannes van Brederode, Christian Alzheimer, Karl Messlinger

**Affiliations:** 1 Institute of Physiology and Pathophysiology, Friedrich-Alexander-University Erlangen-Nürnberg, 91054 Erlangen, Germany; fang.zheng@fau.de (F.Z.); nixdorf-bergweiler@t-online.de (B.E.N.-B.); hansvb@uw.edu (J.v.B.); christian.alzheimer@fau.de (C.A.); 2 Department of Physiology & Biophysics, University of Washington, Seattle, WA 98125, USA

**Keywords:** spinal trigeminal nucleus caudalis, calcitonin gene-related peptide, cell excitability, excitatory postsynaptic currents, migraine

## Abstract

The neuromodulator calcitonin gene-related peptide (CGRP) is known to facilitate nociceptive transmission in the superficial laminae of the spinal trigeminal nucleus caudalis (Sp5C). The central effects of CGRP in the Sp5C are very likely to contribute to the activation of central nociceptive pathways leading to attacks of severe headaches like migraine. To examine the potential impacts of CGRP on laminae I/II neurons at cellular and synaptic levels, we performed whole-cell patch-clamp recordings in juvenile mouse brainstem slices. First, we tested the effect of CGRP on cell excitability, focusing on neurons with tonically firing action potentials upon depolarizing current injection. CGRP (100 nM) enhanced tonic discharges together with membrane depolarization, an excitatory effect that was significantly reduced when the fast synaptic transmissions were pharmacologically blocked. However, CGRP at 500 nM was capable of exciting the functionally isolated cells, in a nifedipine-sensitive manner, indicating its direct effect on membrane intrinsic properties. In voltage-clamped cells, 100 nM CGRP effectively increased the frequency of excitatory synaptic inputs, suggesting its preferential presynaptic effect. Both CGRP-induced changes in cell excitability and synaptic drives were prevented by the CGRP receptor inhibitor BIBN 4096BS. Our data provide evidence that CGRP increases neuronal activity in Sp5C superficial laminae by dose-dependently promoting excitatory synaptic drive and directly enhancing cell intrinsic properties. We propose that the combination of such pre- and postsynaptic actions of CGRP might underlie its facilitation in nociceptive transmission in situations like migraine with elevated CGRP levels.

## 1. Introduction

Calcitonin gene-related peptide (CGRP) is produced by a considerable proportion of primary afferent neurons of the trigeminal and spinal sensory ganglia [1,2,3]. CGRP seems to be further enriched in trigeminal afferents that innervate intracranial structures like meninges and cerebral blood vessels [4], structures which have long been regarded as sources for the generation of headaches [5]. The peptidergic afferents are attributed a nociceptive function, since they mostly co-express nociceptive transducer channels of the transient receptor potential (TRP) family such as TRPV1 or TRPA1 [6]. CGRP is particularly important as a nociceptive mediator in the intracranial pain producing system. Infusion of CGRP induces headaches in patients suffering from migraine but does not cause any other pain [7,8], and CGRP receptor inhibition has been shown to be specifically therapeutic in primary headaches like migraine [9,10]. Inactivation of CGRP or CGRP receptors by monoclonal antibodies also promises to be extremely effective in preventing frequent migraine attacks [11].

Release of CGRP from peripheral endings of peptidergic afferents is involved in neurogenic vasodilatation, the significance of which, for headache generation, is still controversial [12]. However, CGRP release from central terminals of these afferents in the trigeminal and spinal dorsal horn is an established principle in nociceptive neuromodulation [13,14,15]. Microiontophoretic application of CGRP into the trigeminocervical complex in cats increased the activity of second order neurons with intracranial afferent input [16]. In a slice preparation of cervical segments, super-fusion of high concentration of CGRP (500 nM) caused shortening in latency and increased the discharge frequency to current injection in ‘delayed response type’ neurons located in superficial laminae of juvenile rat trigeminal nucleus caudalis (also called Sp5C) [17]. Whether other types of neurons in the superficial Sp5C are also responding to CGRP has not been examined so far.

We focused our study on laminae I and II, because this area has been shown to be particularly enriched by CGRP [3,18]. CGRP receptor components show a strong immunohistochemical signal on primary afferent terminals (i.e., presynaptic location) but there is conflicting evidence for an additional expression on second order neurons (i.e., postsynaptic location) [3,19]. The precise CGRP actions in synaptic transmission within the Sp5C are unclear; however, the involvement of glutamate has been shown in various model systems [20,21,22]. Thus, we aimed to examine whether the effects of CGRP in the in vitro slice preparation are linked to glutamatergic transmission. Indeed, we found that CGRP exerts tonic control over excitatory drive onto Sp5C laminae I/II neurons in normal condition, and further facilitates excitatory transmission, and ‘winds’ postsynaptic neuronal excitability up when CGRP level rises.

## 2. Results

### 2.1. Firing Patterns of Sp5C Laminae I/II Neurons

**Figure 1:**
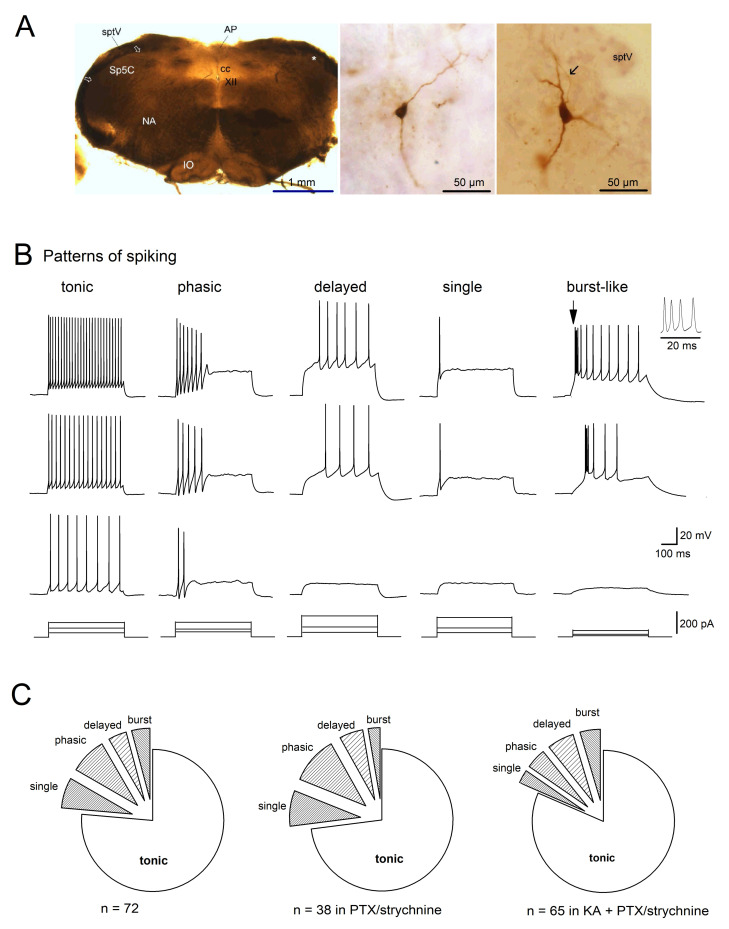
Firing patterns of Sp5C laminae I/II cells. (**A**) Bright field micrographs illustrate a typical freshly prepared transverse slice (350 µm thickness; left), and examples of biocytin-filled neurons in lamina II (middle) and in lamina I (right, with soma location indicated by an asterisk on the left, and axon pointed by arrow). AP, area postrema; cc, central canal; IO, inferior olivary complex; NA, nucleus ambiguous; sptV, spinal tract of the trigeminal nerve; XII, hypoglossal nucleus. (**B**) Five classes of Sp5C laminae I/II cells are distinguished by their properties of action potential discharge to depolarizing currents. Cells were recorded under whole-cell current-clamp mode, with membrane potential initially held at −70 mV (by current injection). Pattern of spiking was determined by their responses (top three panels) to the serial of current injection (bottom panels). The initial burst-like spiking (arrow) was enlarged on the right. (**C**) Distribution of spiking patterns of Sp5C cells recorded in the absence and in the presence of inhibitory and excitatory synaptic blockers. Kynurenic acid (KA, 2 mM) was used to block fast glutamatergic synaptic transmission, and picrotoxin (PTX, 100 µM) plus strychnine (10 µM) were used to block fast GABAergic and glycinergic synaptic transmissions, respectively.

Whole-cell current-clamp recordings were made in neurons located in rich CGRP-immunoreactive lamina I/II of Sp5C from juvenile mouse medullary slices. Two examples of biocytin-filled cells are illustrated in Figure 1A, characterized by more or less pyramidal-shaped somata with few primary dendrites. This neuronal morphology has also been reported among other types present in superficial lamina I in rat Sp5C [23], while mouse multipolar neurons in lamina II are more dominant, as reported by Davies and North [24]. The recorded cells with different electrophysiological profiles had a membrane capacitance of 44.55 ± 2.16 pF (ranging from 19 to 110 pF; *n* = 72) and input resistance (R_N_) of 472.98 ± 35.40 MΩ (ranging from 155 to 1200 MΩ) at −70 mV. Most of these neurons were not spontaneously active and had a resting membrane potential of −65.90 ± 0.77 mV (*n* = 61). When the membrane potential of Sp5C laminae I/II neurons was held at −70 mV, depolarizing current pulses (20–200 pA for 500 ms) elicited action potentials (APs) with characteristic discharge patterns, namely tonic, phasic and delayed spikes, or single spike (Figure 1B), consistent with previous studies [23,24,25,26]. Interestingly, we identified a fifth class of Sp5C neurons that responded to depolarizing pulses in a burst-like manner. As shown in Figure 1B, tonic spiking cells were easily excitable and fired APs regularly throughout the depolarizing current pulse (*n* = 55). Phasic spiking cells fired APs only at the beginning of the supra-threshold stimulus (*n* = 6). Delayed spiking cells never fired APs right at the beginning of the stimulus (*n* = 3). Single spike cells responded with only one AP to depolarizing pulses, even at increased current injections (*n* = 5). Burst-like spiking cells responded to depolarizing current injection by firing a brief burst (3–4 densely packed spikes) or double spikes at the beginning of the stimulus, followed by regular spiking (*n* = 3). Except those with single spike, neurons increased their spiking frequency with increasing stimuli.

Clearly, 55 cells with tonic spiking were dominant among mouse Sp5C laminae I/II neuronal population (76.39%), followed by phasic (8.33%), single (6.94%), delayed (4.17%) and burst-like (4.17%) spiking (Figure 1C; left circle). To figure out whether the diverse discharging patterns of Sp5C laminae I/II are derived from cells intrinsically or influenced by neural network via synaptic connections, we perfused the slices with artificial cerebrospinal fluid (aCSF) containing antagonists for inhibitory GABA_A_Rs (picrotoxin; PTX) and GlyRs (strychnine), and/or together with antagonist for ionotropic GluRs (kynurenic acid; KA). Similar patterns of spiking were observed when inhibitory network impact was removed (Figure 1C, middle circle), with the majority of them firing tonic spikes (27 out of 38 cells, i.e., 71.05%), just like those cells embedded in the intact neuronal network. When the excitatory synaptic transmission was further blocked, the dominance of tonic spiking cells in Sp5C laminae I/II persisted (53 out of 65 cells, i.e., 81.54%; Figure 1C, right circle). The preserved patterns of spiking among the functionally isolated cells suggest the diverse discharges as intrinsic properties of Sp5C cells.

### 2.2. CGRP Excites Tonic Spiking Neurons in Sp5C Laminae I/II

**Figure 2:**
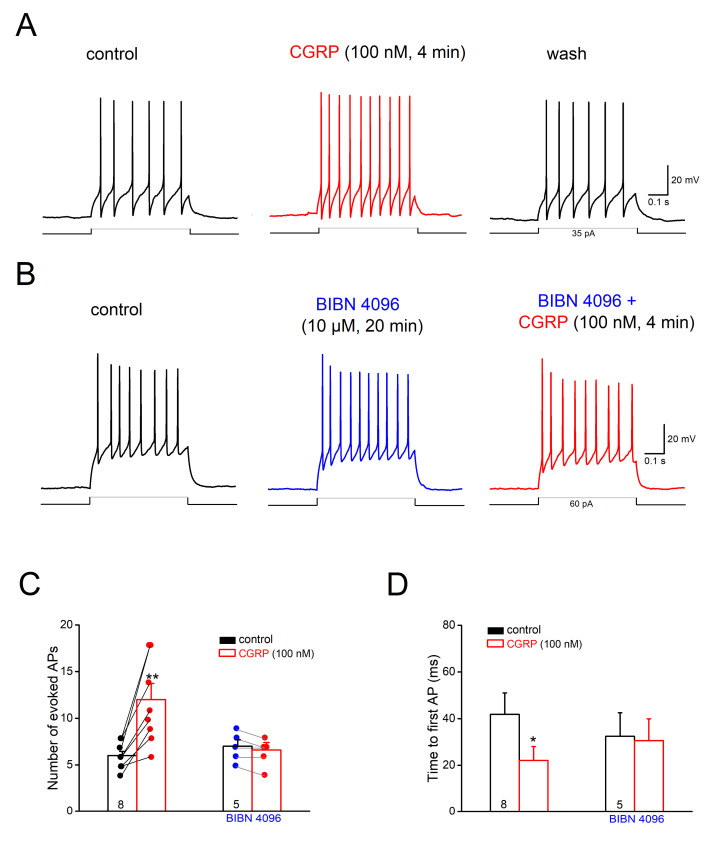
Calcitonin gene-related peptide (CGRP) excites tonic-firing cells in Sp5C laminae I/II. Depolarizing current (35–100 pA) was adjusted individually to evoke 4–10 APs per pulse (500 ms) in each cell before CGRP application, with membrane potential set at −70 mV. (**A**) Original traces taken before, during and after CGRP (100 nM for 5 min) illustrate the reversible enhancement of AP firing in a lamina I cell. (**B**) Representative traces from another cell show how the CGRP receptor antagonist BIBN 4096BS (10 µM) prevents the excitatory effect of CGRP. (**C**,**D**) Histograms summarize CGRP-induced changes in AP discharges (**C**) and time to first AP (**D**) in the absence and in the presence of BIBN 4096BS. Numbers in columns indicate sample size. * *p* < 0.05; ** *p* < 0.01.

Considering neurons with tonic spiking prevailing in Sp5C superficial laminae, we focused on this group of neurons and investigated how CGRP may affect their excitability. AP discharges to depolarizing pulses (500 ms) were adjusted individually to evoke 4–10 APs per pulse in control condition, with membrane potential set to −70 mV. Perfusion of CGRP (100 nM) for 4–8 min increased the AP discharges per pulse from 6.13 ± 0.52 to 11.75 ± 1.59 (*n* = 8; *p* = 0.002, paired *t*-test), and concomitantly reduced the time to the 1st AP from 41.80 ± 9.22 ms to 21.10 ± 5.92 ms (*p* = 0.015, paired *t*-test) (Figure 2A,C,D), together with slight membrane depolarization (2.30 ± 0.42 mV). Such excitatory effect of CGRP on cell firing was reversible (Figure 2A) and was accompanied by an increase in R_N_ (control, 310.17 ± 49.18 MΩ; CGRP, 376.33 ± 54.10 MΩ; *p* = 0.001). When the non-peptide antagonist for CGRP receptors, BIBN 4096BS (also called olcegepant; 10 µM), was applied 17–20 min before CGRP, no change in cell excitability was observed following CGRP (*n* = 5; APs from 7.00 ± 0.71 to 6.60 ± 0.80, *p* = 0.178; Figure 2B–D).

**Figure 3:**
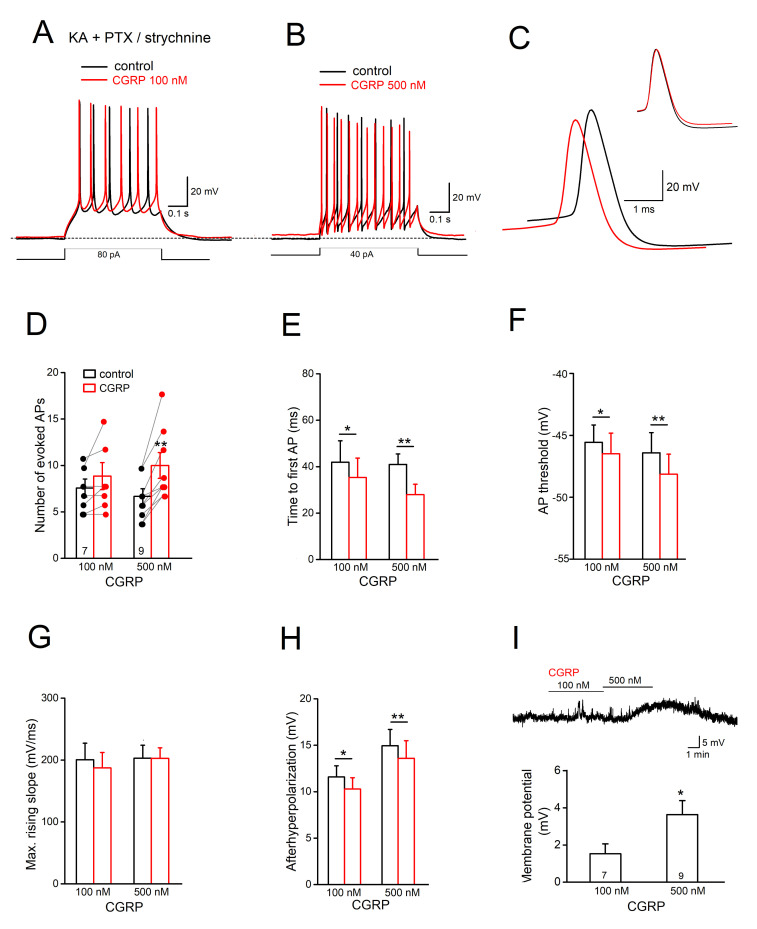
CGRP enhances Sp5C cell intrinsic excitability. Action potentials of Sp5C laminae I/II cells were evoked in the presence of kynurenic acid (KA), picrotoxin (PTX) and strychnine to block fast synaptic transmissions. (**A**,**B**) Overlapped traces from two cells illustrate the concentration-dependent effect of CGRP application on the evoked APs. Dashed line indicates the initial potential of −70 mV. (**C**) Superimposed traces are the 1st APs of the cell in B, with enlarged time scale to illustrate the CGRP-induced reduction in AP threshold and afterhyperpolarization (AHP). (**D**–**H**) Histograms characterize the impacts of CGRP on AP discharges (**D**) and time to evoke 1st AP (**E**), AP threshold (**F**), maximal rising slope of AP (indicative of available sodium channels; (**G**) and AHP (**H**). (**I**) Summary of CGRP-induced membrane depolarization depicted further by a voltage trace above the histogram. * *p* < 0.05; ** *p* < 0.01.

The pronounced excitatory effect of CGRP on Sp5C I/II cells may have many origins, including interplaying of the synaptic inputs and the direct impact on somatic excitability. To decipher the potential loci of actions, we removed all the fast synaptic transmissions by adding a cocktail consisting of antagonists with KA, PTX and strychnine. In the first set of functionally isolated cells (*n* = 7), enhancement in cell firing was dramatically reduced, with only four cells responding to 100 nM CGRP with slight increase in AP number and no change in the remaining cells (Figure 3A,D). When we normalized the change of AP number as percentage of control values, 100 nM CGRP induced an increment of 128.11 ± 8.22% in these four input-deprived cells, which is significantly less than that in control conditions with normal synaptic inputs (*n* = 8, 189.02 ± 15.56%; Figure 2C; *p* = 0.025). Although CGRP could slightly shorten the time to 1st AP (*n* = 7, from 41.93 ± 9.19 ms to 35.33 ± 8.36 ms; *p* = 0.019, paired *t*-test) (Figure 3E), such overall attenuation of CGRP’s facilitating effect on tonic firing after removal of synaptic inputs suggests the potential contribution of the synaptic network. However, when CGRP concentration was raised to 500 nM, as tested with the isolated delayed spiking neurons in Sp5C laminae I/II [17], its direct effect on the membrane intrinsic properties became clear, with constant excitation in the tonic spiking neurons (*n* = 9; Figure 3B,D). Accompanied by membrane depolarization of 3.64 ± 0.75 mV (*p* = 0.040 vs. 100 nM 1.53 ± 0.53 mV; Figure 3I), 500 nM CGRP increased APs (from 6.55 ± 0.75 to 9.88 ± 1.31 per pulse; *n* = 9; *p* = 0.002, paired *t*-test) and decreased the time to the first AP (from 40.98 ± 4.49 ms to 27.94 ± 4.49 ms; *p* = 0.001, paired *t*-test) in these isolated tonic spiking neurons (Figure 3D,E), which essentially mimics the changes induced by 100 nM CGRP with intact synaptic drives (Figure 2).

To answer the question of how CGRP may affect the somatic intrinsic properties, we performed further analysis of AP parameters. As shown in an example in Figure 3C, superposition of first APs before and during CGRP suggests that 500 nM CGRP lowered the threshold for AP generation from −46.41 ± 1.08 mV to −48.14 ± 1.24 mV (*p* = 0.002, paired t-test; Figure 3F). However, 500 nM CGRP did not affect AP amplitude (from 62.04 ± 2.71 mV to 62.29 ± 2.55 mV, *p* = 0.760) and its maximal rising slope (from 203.08 ± 20.792 mV/ms to 202.79 ± 17.06 mV/ms, *p* = 0.960; Figure 3G), which is an indicator of the available sodium channels. While the half-width of AP was not affected by 500 nM CGRP (from 0.92 ± 0.09 ms to 0.93 ± 0.98 ms, *p* = 0.461), the size of afterhyperpolarization (AHP), determined as the difference between voltage threshold and AHP peak, was slightly but significantly reduced from 14.95 ± 1.76 mV to 13.59 ± 1.91 mV (*p* = 0.005, paired t-test; aligned in Figure 3C; Figure 3H). Interestingly, detectable changes in AP threshold and AHP were also observed with 100 nM CGRP (Figure 3F,H; Table 1).

**Figure 4:**
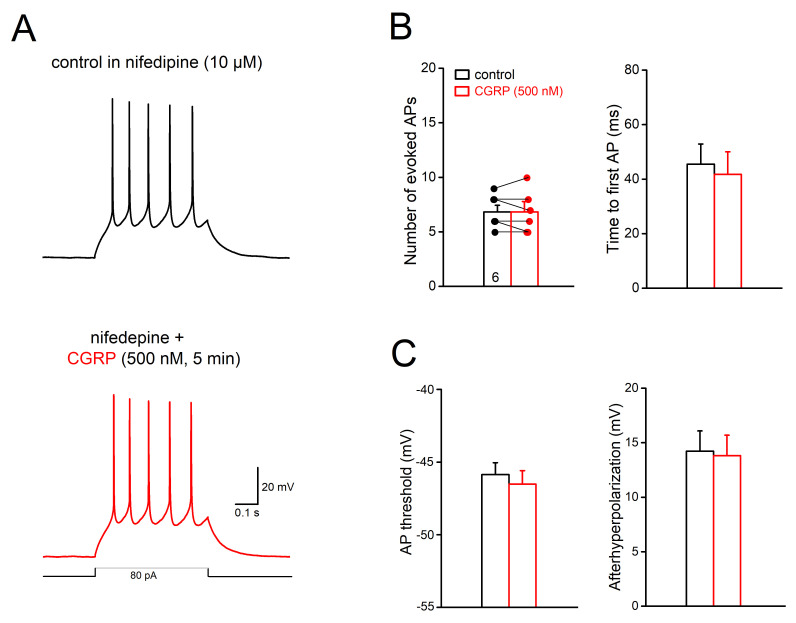
Involvement of L-type calcium channels in the excitatory effect of CGRP. All recordings were performed in the cocktail of KA, PTX and strychnine to block fast synaptic transmissions. (**A**) Voltage traces were collected from a Sp5C cell in laminae II before and during CGRP (500 nM) application, in the presence of the L-type calcium channel blocker nifedipine (10 µM). (**B**,**C**) Histograms summarize that nifedipine blocks the responses of Sp5C cells to high concentration of CGRP (500 nM), manifested in AP discharges and time to evoke 1st AP (**B**), voltage threshold for AP and afterhyperpolarization (**C**).

Which ion channel(s) might be modulated by CGRP to enhance intrinsic firing? A potential candidate are voltage-dependent calcium channels, as therapeutic effects of calcium channel antagonists have long been noted in treating migraine [27,28,29]. Here we probed the L-type calcium channels, which have been suggested to influence spike generation [30] and are essential for windup of spinal dorsal horn neuronal discharges, a cellular mechanism proposed for sensitization in pain [31]. In the presence of the excitatory and inhibitory synaptic blockers, high concentration of CGRP (500 nM) applied to slices treated with L-type calcium channel blocker nifedipine (10 µM, 15–40 min) failed to change tonic AP discharge (*n* = 6; control 6.83 ± 0.60, 500 nM CGRP, 6.83 ± 0.79, *p* = 1.00; Figure 4A,B) and membrane potential (0.05 ± 0.77 mV). When we compared the 1st AP in response to the depolarizing pulse, no significant changes in AP threshold and AHP were observed in most of the test cells (4 out of 6 cells) (threshold: control −45.86 ± 0.82 mV vs. 500 nM CGRP −46.51 ± 0.92 mV, *n* = 6, *p* = 0.086; AHP: control 14.23 ± 1.85 mV vs. 500 nM CGRP 13.82 ± 1.87 mV, *p* = 0.109; *n* = 6, Figure 4C). As expected, 500 nM CGRP together with the calcium channel blocker did not affect the amplitude, maximal slope of rising phase and half-width of AP (data not shown). Note that in a few cells (*n* = 5), we monitored the effect of nifedipine alone (10 µM) and found that nifedipine slightly reduced the tonic cell firing (from 7.00 ± 0.55 per pulse to 5.60 ± 0.68 at 5 min, *p* = 0.021, paired *t*-test).

### 2.3. CGRP Facilitates Excitatory Synaptic Inputs onto Laminae I/II Neurons

**Figure 5:**
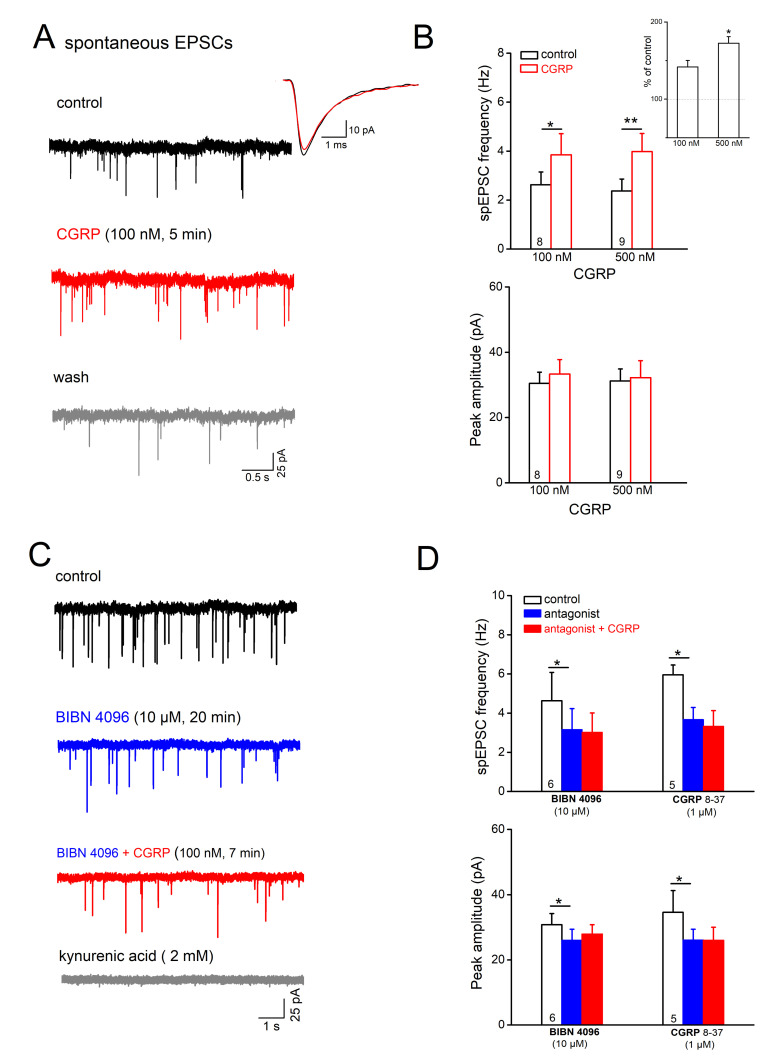
Calcitonin gene-related peptide (CGRP) facilitates excitatory synaptic drive onto Sp5C laminae I/II cells. Spontaneously occurring excitatory postsynaptic currents (spEPSCs) were monitored under whole-cell voltage-clamp mode at −70 mV, in the presence of antagonists for GABA_A_Rs and GlyRs. (**A**) Raw traces of spEPSCs from a lamina I cell were collected before CGRP application (control), 5 min in CGRP (100 nM) and 10 min after wash. Superimposed traces on the right represent the averaged events from the cell before (black trace) and during drug application (red trace). (**B**) Histograms summarize the effects of acutely applied CGRP on the frequency and the averaged peak amplitude of spEPSCs. Inset in up-right corner with the normalized changes in spEPSC frequency further reinforces the dose-dependent effect of CGRP. (**C**) Typical responses of spEPSCs to CGRP (100 nM) in the presence of a potent non-peptide antagonist BIBN 4096BS (10 µM, 20 min). Kynurenic acid (KA, 2 mM) was applied at the end of this experiment to verify the glutamatergic origin of spEPSCs. (**D**) Summary of the dampening effects of CGRP receptor antagonists on spEPSCs in Sp5C I/II cells. * *p* < 0.05; ** *p* < 0.01.

The robust enhancement of cell excitability after application of 100 nM CGRP in intact slices (Figure 2), but not in synaptically silenced slices (Figure 3), made us wonder whether the peptide drives predominantly excitatory input onto Sp5C neurons to augment their firing. To investigate how CGRP modulates the excitatory synaptic inputs onto Sp5C cells, we performed whole-cell voltage-clamp recordings from Sp5C cells (clamped at −70 mV) and monitored the spontaneously occurring excitatory postsynaptic currents (spEPSCs; Figure 5), in the presence of GABA_A_ receptor antagonist PTX (100 µM) and GlyR antagonist strychnine (10 µM). Application of CGRP at 100 nM significantly increased the frequency of spEPSCs from 2.63 ± 0.52 Hz to 3.85 ± 0.86 Hz (*n* = 8; *p* = 0.015, paired *t*-test; Figure 5A,B). Such facilitating effect of CGRP on excitatory synaptic transmission was reversible upon wash (Figure 5A). When we normalized the change as percentage of control values, 100 nM CGRP produced an increment of 41.82 ± 8.43% in spEPSC frequency. In contrast to consistent enhancement of EPSC frequency, a change in EPSC amplitude in response to 100 nM CGRP was only observed in few cells (3 out of 8 cells). The overall not significant change in the averaged peak amplitudes of spEPSCs (control, 30.50 ± 3.41 pA vs. CGRP 100 nM, 33.32 ± 4.43 pA, *n* = 8, *p* = 0.334; Figure 5B) points to the presynaptic site as primary target for CGRP. This notion was further reinforced when higher concentration of CGRP was used. Indeed, 500 nM CGRP dramatically enhanced spEPSC frequency by 68.80 ± 8.27% (*n* = 9, Figure 5B, inset), which is much stronger than that of 100 nM CGRP (*p* = 0.038). Again, averaged peak amplitude of spEPSCs was generally not altered following 500 nM CGRP (only in two out of nine cells; Figure 5B). Further analysis of averaged EPSC traces revealed that 100 nM CGRP had no appreciable effect on the kinetics, including 10–90% rising time (*n* = 8; control, 0.35 ± 0.03 ms; 100 nM CGRP, 0.36 ± 0.03 ms; *p* = 0.500) and decay tau (control, 1.63 ± 0.21 ms; 100 nM CGRP, 1.73 ± 0.23 ms; *p* = 0.251). However, 500 nM CGRP tended to prolong both the rising and decaying phases (*n* = 9; rising time from 0.45 ± 0.04 ms to 0.49 ± 0.05 ms, *p* = 0.008; and decay tau from 1.94 ± 0.19 ms to 2.18 ± 0.22 ms, *p* = 0.050).

Such a facilitating effect of CGRP on spEPSCs was abrogated when we repeated the experiments in the presence of CGRP receptor peptide and non-peptide antagonists (Figure 5C,D). Interestingly, we observed that these antagonists alone reduced spEPSC frequency and peak amplitude (Figure 5C,D), suggesting a strong tonic control of CGRP over glutamate release. In this context, BIBN 4096BS (10 µM) alone reduced the frequency from 4.71 ± 1.42 Hz to 3.16 ± 1.07 Hz (*n* = 7 for 15–20 min, *p* = 0.012, paired *t*-test), and decreased their averaged peak amplitude (from 30.80 ± 3.42 Hz to 25.99 ± 3.44 Hz, *p* = 0.046). Comparable suppressive effects on spEPSCs in Sp5C I/II cells were also observed with the peptide antagonist CGRP 8–37 (1 µM for 8–16 min; *n* = 5). Further application of CGRP at 100 nM to these pre-treated slices failed to enhance spEPSCs in Sp5C cells. For example, the frequency of spEPSC was 3.05 ± 0.91 Hz in BIBN 4096BS (*n* = 6) and 2.82 ± 0.87 Hz in CGRP plus BIBN 4096BS (*p* = 0.122, paired *t*-test; Figure 5C,D).

### 2.4. CGRP Receptor Immunofluorescence in Sp5C Laminae I/II

**Figure 6:**
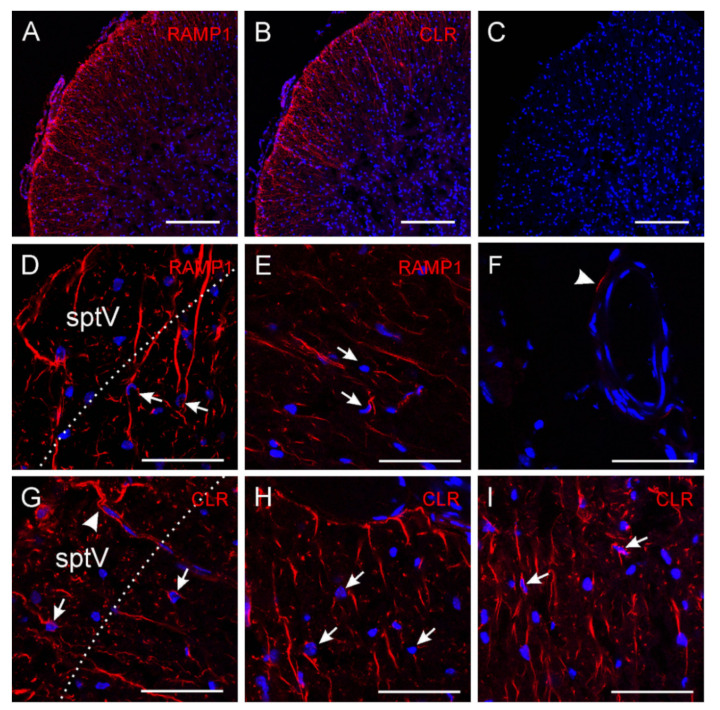
Immunohistochemically processed sections showing the outer laminae of the mouse dorsolateral Sp5C. Immunofluorescence of CGRP receptor components receptor activity modifying protein 1 (RAMP1) (**A**,**D**,**E**) and calcitonin-like receptor (CLR) (**B**,**G**–**I**) in the spinal trigeminal tract (sptV) and lamina I/II of the mouse Sp5C ((**A**–**C**,**G**) dorsolateral, (**D**,**I**) ventrolateral, (**E**,**H**) lateral); (**C**,**F**) are control stainings without first antibody but incubated with the second antibody Cy3; blue is nucleus staining (DAPI). The dotted lines in (**D**,**G**) show the approximate border between sptV and lamina (**I**). Arrows point to cell bodies that are closely approached by RAMP1/CLR immuno-positive nerve fibres, the arrowhead in G shows CLR immuno-positive fibres accompanying a penetrating medullary blood vessel, and the arrowhead in the control staining F points to an unspecific immunofluorescence in the wall of a blood vessel; magnification bars 200 µm in (**A**–**C**) and 50 µm in (**D**–**I**). No CGRP receptor component immunofluorescence of cell bodies is visible.

To provide the morphological basis for the above pre- and post-synaptic functional changes following CGRP, we performed immunostainings for CGRP receptors. CGRP receptors are composed of three proteins, calcitonin-like receptor (CLR) with seven transmembrane spanning domains, the receptor activity modifying protein 1 (RAMP1) with one transmembrane domain and an additional intracellular protein, the receptor component protein (RCP) [32,33]. These three elements are required to form a functional CGRP receptor. We used antibodies against CLR and RAMP1 linked to fluorescing Cy3 to visualize the CGRP receptor proteins. In addition, DAPI was used as nucleus staining to localize cells. As shown in Figure 6, CLR and RAMP1 immunoreactivity was observed in cross- and oblique-sectioned fibres in the spinal trigeminal tract and lamina I/II of the spinal trigeminal nucleus, supporting the presynaptic loci of CGRP receptors on terminals of primary afferents. Occasionally immunoreactivity along small blood vessels was seen. Some pial blood vessels outside the medulla also showed immunoreactivity in medial or endothelial location. However, no immunoreactivity was detected in cell bodies visible by DAPI staining but cells were frequently approached by fibre endings resembling synaptic contacts. Omitting primary antibodies did not yield any CLR or RAMP1 immunoreactivity apart from very few unspecific stainings.

## 3. Discussion

In the present study we took advantage of an in vitro slice preparation and identified the tonic control of CGRP over the excitatory drive onto Sp5C laminae I/II neurons in normal condition, and the concerted boosting effects of elevated CGRP level on the synaptic transmission and activity of second order neurons. Our results from synaptic and cellular levels confirm the concept that CGRP acts as a positive neuromodulator in the Sp5C, suggested from in vivo studies that iontophoretic application of CGRP into the trigeminocervical complex increased the activity of second order neurons upon peripheral stimulation or glutamate application, and local or systemic application of its antagonists caused the reverse effect [16,34,35]. CGRP is regarded as a key mediator in the generation of migraine and other attacks of primary vascular headaches [14,36]. This is mainly based on clinical observations, including increased CGRP plasma levels during migraine attacks [37,38] (but see also [39]), delayed headache attacks after CGRP infusion in patients suffering from primary headaches [7], and the effective treatment of migraine with CGRP receptor antagonists (like olcegepant/gepants; [40,41,42] and monoclonal antibodies targeting CGRP or its receptor [43]. However, the central action of these antagonists and antibodies remains unclear, as they cannot readily penetrate the blood–brain barrier [44,45,46].

### 3.1. Classification of Sp5C Laminae I/II Neurons

We focused our study on the superficial laminae I and II (preferentially IIo) of the Sp5C as the specific nociceptive transmission sites, which are particularly enriched by CGRP [18]. Neurons in the superficial laminae of Sp5C are known to be composed of heterogeneous groups with diverse morphological and electrophysiological properties. Using whole-cell recordings from laminae I/II neurons in transverse brainstem slices of juvenile C57BL/6 mice, our classification of Sp5C superficial neurons regarding their response characteristics as action potentials conformed well to those from the literature in in vitro preparations from rats [23,25] and mice [24,26]. Among the patterns of tonic, phasic, delayed and single spiking that have been well-described in P14–35 rodent slices, we have sampled a prevalent population with tonic spiking among 175 neurons in P8–14 mice (77%). In addition, we recorded a small group of neurons (4%) responding to depolarizing current pulses in a burst-like manner. A burst firing pattern that closely resembles those observed in our study was reported by Ruscheweyh et al. [47] in spinal dorsal horn lamina I neurons of rats. Interestingly, a burst of spike discharges in the Sp5C was observed in a slice preparation with intact peripheral nerves attached in response to peripheral nerve stimulation in neonatal P4–6 rats [48] and in superficial spinal cord neurons [49]. It is possible that burst-like neurons have not been encountered in mice Sp5C in earlier studies because of differences in the age groups used. Taking into account that Sp5C is the largest subdivision of the spinal trigeminal nucleus that consists of an elongated laminated portion merging without clear boundaries with the cervical dorsal horn, regional-specific differences might also account for differences in identifying particular neuronal classes [50,51].

As an experimental model for characterizing central information processing at cellular and synaptic levels, an acute brain slice preparation with largely preserved local structural integrity enables stable recording of visualized cells and accessibility of pharmacological manipulations. While some in vitro slice preparations preserve peripheral nerves for stimulation experiments when recording synaptically activated neurons in Sp5C (e.g., [48]), in our experimental setup we were unaware of the particular input to the patch-clamped neurons. The ongoing activity of Sp5C I/II cells observed in vivo were largely reduced in our transverse slices, partially due to loss of the important longitudinal connection. Only 15–18% of the sampled cells in Sp5C superficial laminae fired action potentials at rest (i.e., 11 out of 72 cells in control setting, 7 out of 38 cells after removal of inhibitory inputs, and 10 out of 65 cells after silence of both excitatory and inhibitory inputs).

### 3.2. CGRP Promotes Neuronal Excitability in Sp5C Superficial Laminae

In the present study, we focused on the effects of CGRP on the most frequently encountered type of neurons, namely those with tonic firing pattern. We found that CGRP at a concentration of 100 nM dramatically enhanced the cell excitability of the tonic spiking cells in laminae I/IIo, by shifting the membrane potential in a depolarizing direction and promoting action potential discharges. Such overall enhancement in responsiveness of Sp5C superficial laminae cells is consistent with the finding in pain-related spinal dorsal horn neurons in rat, where CGRP was found effective at 10 nM in the reduced slice preparation of Bird et al. [52]. To obtain a comparable effectiveness of CGRP in exciting these tonic cells from synaptic silenced slices, a high concentration of CGRP at 500 nM was used according to an earlier study with second-order delayed response type neurons from P14–18 rat Sp5C slices [17], suggesting the recruitment of synaptic networks following CGRP upregulation. In the slice preparation of Bird et al. [52] CGRP facilitated synaptic transmission notably after induction of arthritis that presumably had caused central sensitization including elevated CGRP sensitivity. Apart from this difference it remains speculative whether there are major differences between the spinal and the medullary dorsal horn regarding CGRP effects and their involvement in pain generation in spinal and trigeminal areas. Without knowledge about the connectivity and transmitter identity of the tonic firing neurons we recorded from in the superficial laminae of mouse Sp5C slices, the functional implications of their strong responsiveness to CGRP under physiological and pathological conditions remain to be determined. By taking advantage of genetic reporter lines, Pradier et al. [26] reported recently that tonic firing neurons are among the neuronal population labelled with GABA/glycine vesicular transporter (exclusively inhibitory neurons), but not among that labelled with somatostatin (mostly co-expressed in excitatory neurons).

How can the activation of CGRP signaling be linked to the membrane intrinsic properties? The canonical pathway of CGRP signaling is known to couple to adenylyl cyclase, regulating a wide array of cellular functions via elevated cyclic AMP levels. Our detailed analysis of AP dynamics indicates that CGRP is capable of lowering the voltage threshold for AP generation and reducing the AHP (Figure 3). Both changes could effectively promote cell firing; however, the underlying mechanisms may be distinct and remain elusive. The unaltered upstroke speed and peak amplitude of spike following CGRP argues against a direct action on fast sodium channels. Among the potential ion channel targets of CGRP, we tackled the possible involvement of voltage-gated calcium channels (VGCCs), which are activated by membrane potential depolarization and cause calcium influx and consequently calcium-dependent events. VGCC antagonists (mostly L-type) were introduced in preventing migraine because of their wide effects [29]. All types of VGCCs (*p*/Q-, N-, L- and T-type) are abundantly found in rodent Sp5C, and only the cocktail of blockers in conjunction of removal of extracellular calcium are able to affect CGRP release following high potassium challenge [53]. In addition to promoting CGRP release from nerve terminals, the widely distributed VGCCS may also underlie the calcium currents in superficial laminae neurons [24,54]. CGRP has been shown to facilitate calcium currents in neurons in nucleus tractus solitarius, mainly via L-type VGCCs [55]. The dihydropyridine-sensitive L-type calcium channels have been considered suitable to impact cell firing regarding their kinetics [30], and indeed, they are crucial for windup of neuronal discharges (‘sensitization’) in spinal dorsal horn [56]. Exceeding our expectations by far, pre-treating Sp5C superficial neurons with nifedipine successfully blocked the effects of CGRP on membrane potential, tonic discharges, threshold for spike initiation and AHP (Figure 4). A potential link between L-type channels and AHP might lie on calcium influx through these channels during membrane depolarization, which may trigger certain potassium channels to affect the AP repolarizing process. For example, activation of large (big)-conductance calcium-activated potassium channels (BKCa) in the Sp5C affects cell firing [57], and has been implicated in headache [58,59]. Clearly the present results warrant further detailed examinations to clarify the underlying ionic mechanisms of CGRP. Nevertheless, taken together with VGCC antagonists-mediated inhibition of CGRP release [53], the ability of calcium channel blockers to brake CGRP stimulation on second order neurons in Sp5C (present study) may contribute, at least partially, to their migraine preventive effect.

### 3.3. CGRP Enhances Glutamatergic Transmission in Sp5C Superficial Laminae

Projection from the trigeminal ganglion to the trigeminal nucleus is involved in orofacial and craniofacial pain, which preferentially takes place in superficial laminae I-II and partly in the deeper laminae V-VI [60,61]. Excitatory drives onto the Sp5C superficial neurons have been detected in few studies [23,24,25,62]. More recently, however, an elegant study using optogenetic tools was able to selectively activate a nociceptor-enriched subset of primary afferents and characterized long-term plasticity at excitatory synapses onto second-order neurons in superficial laminae of the trigeminal nucleus [26]. The presence of glutamate vesicles in the presynaptic nerve terminals [63] and the strong CGRP immunoreactivity of nerve fibers in the superficial laminae of Sp5C [3] argue for a cooperative effect of this neuromodulator and glutamate in synaptic transmission. This perspective view is supported by functional work in our laboratory where glutamate receptor blockade reduced CGRP release in brainstem slices [15]. By monitoring the overall spontaneous EPSCs in laminae I/II neurons, which may encompass origins of central trigeminal terminals as well as local excitatory network, here we provide further evidence that CGRP promotes glutamate release in a dose-dependent manner. Interestingly, we also reveal a tonic control of CGRP over excitatory neurotransmitter release in normal conditions, in that the CGRP receptor peptide and non-peptide antagonists decrease the frequency and size of EPSCs in laminae I/II neurons.

### 3.4. Pre- or Postsynaptic Mechanisms of CGRP?

Our and other’s immunohistochemical studies failed to observe CGRP receptor components on second order neuron cell bodies in the Sp5C (present study; [3,64]; still under debate, see [65]). The preferential presynaptic action of CGRP is well supported by the intense immunostaining of CGRP and its receptors on primary terminals and by our functional readouts that 100 nM CGRP promotes both synaptic transmission and neuronal excitability, whereas the same concentration is less effective when fast synaptic transmission is blocked. However, the fact that under these conditions of functional isolation a higher concentration of CGRP (500 nM) showed excitatory effects on tonic spiking neurons argues for an additional direct effect of CGRP on these cells. It is a matter of speculation by which mechanism this effect occurs. The amylin-1 receptor is regarded as a second CGRP receptor, which binds CGRP at a similar affinity as the canonical CGRP receptor and is also coupled to adenylyl cyclase activation, whereas the downstream intracellular effects are not identical [66]. However, it is not very likely that CGRP acts on postsynaptic amylin-1 receptors, because they are composed of the calcitonin receptor protein and the RAMP1 component [67], which we could not identify immunohistochemically on Sp5C neurons. Other receptors of the calcitonin family, such as adrenomedullin receptors, which also have a significantly lower affinity to CGRP, are not likely to come into account, because they contain the CLR compound of the CGRP receptors [67], which was again not detected on neuronal cell bodies. It is also conceivable that the weak CGRP receptors on postsynaptic neurons may escape the detection of the light/confocal microscope for antibody immunofluorescence. Further electron microscopy study might clarify such a possibility.

Our finding that, in addition to its presynaptic actions, CGRP at a five-fold concentration has additional excitatory effects directly on second-order neurons may be important in terms of central sensitization as a hypothetical mechanism involved in severe headache attacks [68]. We speculate that this second stage of activation can be caused by an increased release of CGRP from central terminals of vigorously activated primary afferents. Thereby, CGRP may spill over to second-order neurons not directly contacted by the CGRP-releasing afferents, which could be a mechanism explaining phenomena like hyperalgesia and allodynia during severe headaches like migraine [68].

## 4. Materials and Methods

### 4.1. Animals and Trigeminal Nucleus Slice Preparation

All experiments and procedures were carried out according to the German guidelines and regulations of care and treatment of laboratory animals and the European Communities Council Directive of 24 November 1986 (86/609/EEC), amended 22 September 2010 (2010/63/EU). Species and numbers of animals used were reported at the end of each experimental year to the administration for animal protection at the Friedrich-Alexander-University Erlangen-Nürnberg. C57BL/6J mice (postnatal 8 to 14 days) of either sex were used in the present study. They were bred in our animal facilities and housed under 12:12 light:dark cycles with free access to food and water. Mice were decapitated under sevoflurane anesthesia and the brains were rapidly removed and immersed in ice-cold high-sucrose solution containing (in mM): 75 sucrose, 87 NaCl, 2.5 KCl, 7 MgCl_2_, 0.5 CaCl_2_, 1.25 NaH_2_PO_4_, 25 NaHCO_3_ and 30 D-glucose, saturated with 95% O_2_ and 5% CO_2_ (pH 7.4). Serial transverse slices (350 µm) were collected from the medulla oblongata caudally to the obex using a vibro-slice (VT1200 S, Leica Biosystems Nussloch GmbH) and incubated in the same solution at 35 °C for 10 min. The slices were then kept at room temperature in artificial cerebrospinal fluid (aCSF) containing (in mM): 125 NaCl, 2.5 KCl, 3 MgCl_2_, 1 CaCl_2_, 1.25 NaH_2_PO_4_, 25 NaHCO_3_ and 30 D-glucose, at least 2 h before they were transferred into the recording chamber that was mounted on the stage of an upright microscope (BX50WI Olympus).

### 4.2. Electrophysiological Recordings

The outer laminae of the spinal trigeminal nucleus caudalis (Sp5C) approximately 1 mm caudal to the obex was identified according to the Brainstem Atlas C57BL/6 Mouse [69]. The presence of area postrema, central canal, hypoglossal nucleus, caudal part of nucleus ambiguous and the well-established inferior olive guided identification of the Sp5C (Figure 1A, left). Individual neurons of superficial laminae I and II, most of them attributed to the outer part of lamina II (lamina IIo) in the upper half of the Sp5C, were visualized by means of Dodt infrared gradient contrast in conjunction with a contrast-enhanced CCD camera attached to the microscope. Whole-cell recordings of visualized neurons in lamina I/IIo of Sp5C, were performed in aCSF containing (in mM): 125 NaCl, 3 KCl, 1.5 MgCl_2_, 2.5 CaCl_2_, 1.25 NaH_2_PO_4_, 25 NaHCO_3_, 30 D-glucose (pH 7.4) at 30 °C, using a submerged chamber equipped with a gravity-driven perfusion system (flow rate 2~3 mL/min). All solutions were constantly gassed with 95% O_2_ and 5% CO_2_. Patch pipettes were filled with (in mM) 135 potassium gluconate, 5 Hepes, 3 MgCl_2_, 5 EGTA, 2 Na_2_ATP, 0.3 Na_3_GTP and 4 NaCl (pH 7.3). The electrode resistance ranged from 3 to 5 MΩ. Biocytin (1%) was occasionally used for filling and locating the cells.

Action potential (AP) discharge of Sp5C cells were monitored under whole-cell current-clamp mode. Healthy cells in lamina I/II included in this study met our electrophysiological criteria: a membrane input resistance (R_N_) of > 150 MΩ, a stable resting membrane potential more negative than −50 mV, and action potentials with overshoot > 15 mV. To test cell excitability, depolarizing pulses were used to elicit APs at −70 mV by injecting current. In the set of experiments to synaptically isolate the recorded cells, fast synaptic transmissions were blocked by adding the AMPA/NMDA glutamate receptor antagonist kynurenic acid (KA; 2 mM), the GABAA-receptor antagonist picrotoxin (PTX; 100 µM) and the glycinergic receptor antagonist strychnine (10 µM).

To monitor excitatory synaptic inputs, cells were clamped at −70 mV under whole-cell voltage-clamp mode, and spontaneous excitatory postsynaptic currents (spEPSCs) were collected in the presence of PTX and strychnine. All potentials were corrected for liquid junction potential. Signals were filtered at 6 kHz (for action potentials) or 2 kHz (for synaptic currents) and sampled at 20 kHz using a Multiclamp 700B amplifier together with Digidata 1550 interface and pClamp10.6 software (Molecular Devices, Sunnyvale, CA).

Drugs and chemicals were obtained from Tocris (Cologne, Germany) and Sigma (Deisenhofen, Germany). Data analysis was performed off-line with Clampfit 10.6 (Molecular Devices). Spontaneous events were detected using an automated event detection algorithm. At least three recordings (20 s each) collected at various time points were used to quantify the frequency, peak amplitude and kinetics of synaptic inputs. Rising time was measured from 10–90% of the peak response. The decay of averaged currents was fitted with single exponential functions and tau reflects the time required for a spEPSC to decay to 37% of its peak value.

### 4.3. Method for Biocytin Labeling

At the end of experiments with biocytin in the recording pipettes, slices were fixed in 4% paraformaldehyde in 0.1 M phosphate buffer overnight, cryoprotected and sectioned at 60 µm intervals. The cryostat sections were directly mounted onto glass slides and incubated in avidin-biotin conjugated horseradish peroxidase (ABC standard Elite Kit, PK-4000, Vector Lab, Burlingame, CA, USA). For visualizing the tracer, sections were reacted with diaminobenzidine and hydrogen peroxidase (DAB Kit SK-4100, Vector), followed by a very brief counterstaining with Hematoxylin QS (H-3404, Vector). After completely drying, the neurons were either directly embedded in Fluoromount or, after a series of dehydrating steps, cover-slipped with Merckoglas (Merck, Darmstadt, Germany). Pictures were taken with a digital camera ColourView II on a Zeiss Axioscope.

### 4.4. Immunohistochemistry for CGRP Receptors

For immunostaining of CGRP receptor components, adult mice (23–25 g) were euthanized with an intraperitoneal overdose of 150–200 mg/kg thiopental (Trapanal^®^, Byk Gulden, Germany). After quick thoracotomy, the animals were perfused through the left ventricle with warm isotonic saline for about 2 min followed by a solution of 4% paraformaldehyde in 0.1 M phosphate buffer (pH 7.4) for 6–10 min. Following caudal craniotomy, the brainstem together with the caudal third of the cerebellum including medulla oblongata and the first three cervical segments of the spine were carefully dissected, washed in phosphate-buffered saline (PBS, pH 7.4) overnight and stored for one day in 20% buffered sucrose for cryoprotection. The brainstem was mounted on Tissue-Tek (GSV1, Slee Technik, Mainz, Germany), rapidly frozen in methyl-butane at −46 °C and stored at −20 °C. Using a cryostat (Leica Mikrosysteme, Bensheim, Germany), series of 20 µm thick cross-sections were cut from the second cervical segment to the obex of the medulla. Cryostat sections were mounted on poly-L-lysine-coated slides, dried for 1 h at room temperature. The slides were incubated with 5% normal goat serum (Dako, Hamburg, Germany) containing 0.5% Triton X-100 (Merck, Darmstadt, Germany) and 1% bovine serum albumine (BSA; Roth, Karlsruhe, Germany) in PBS for 1 h at room temperature. After rinsing in PBS, the sections were incubated for immunolabelling with polyclonal primary antibodies raised in rabbit against RAMP1 (anti-RAMP1, AA 139–148, 1:100) or CLR (anti-CALCRL, 1:12; Antibodies-Online, Aachen, Germany) diluted in PBS with 1% BSA and 0.5% Triton X-100 at room temperature overnight. After washing with PBS (3 × 5 min) the sections were incubated with goat anti-IgG coupled to indocarbocyanine (Cy3, 1:100; Dianova, Hamburg, Germany) in PBS with 0.5% Triton X-100 and 1% BSA at room temperature. The sections were rinsed again with PBS, mounted with Roti-Mount FluorCare DAPI (4′,6′-diamidino-2-phenylindole hydrochloride, Carl Roth) and cover-slipped. Specificity of the immunocytochemical reactions was tested by omission of primary antibodies and their replacement with PBS with 1% BSA and 0.5% Triton X-100. Confocal imaging was performed with an inverse stage LSM 710 Axio Examiner Z1 microscope (Carl Zeiss) using dry objective lenses (10× and 20× with numerical apertures of 0.3 and 0.8) and a Plan-Apochromat 63× oil DIC objective with a numerical aperture of 1.40. The filter settings of the confocal scanner were 358/461 nm excitation/emission for DAPI and 546/563 excitation for Cy3. Extended focus images and z-series of six optical sections at z-increments of 1.5 µm were created. Images of 512 × 512 pixels were obtained, and the channels of each image were merged into a 12-bit RGB tiff-file using confocal assistant software ZEN 2010 (Carl Zeiss). Adjustment for brightness and contrast was performed. To arrange images and apply text and scale bars, images were imported into Corel Draw Graphics Suite X7 (Corel, Dublin, Ireland).

### 4.5. Statistical Analysis

Data are expressed as means ± SEM. Statistical comparisons of data were performed using ANOVA or Student’s t-test as appropriate after testing normal distribution of data. Significance was assumed for *p* < 0.05.

## Figures and Tables

**Table 1:** Effects of CGRP on the properties of Sp5C superficial laminae cells. Data are presented as mean ± SEM. Except for the resting membrane potential (RMP), membrane properties were characterized based on a holding potential of −70 mV. The first action potential (AP) responding to the depolarizing current injection (500 ms) was used for analysis. * *p* < 0.05; ** *p* < 0.01.

				KA + Picrotoxin +	Strychnine	
Tonic spiking cells	Control (*n* = 8)	CGRP (100 nM)	Control (*n* = 7)	CGRP (100 nM)	Control (*n* = 9)	CGRP (500 nM)
Resting membrane potential (RMP; mV)	−69.08 ± 1.92	-	−66.30 ± 3.27	-	−67.42 ± 1.53	-
Cm (pF)	58.78 ± 7.92	-	53.33 ± 11.07	-	52.50 ± 6.50	-
# of APs per pulse	6.13 ± 0.52	11.75 ± 1.59 **	7.42 ± 0.90	8.71 ± 1.34	6.55 ± 0.75	9.89 ± 1.31 **
Time to 1st AP (ms)	41.80 ± 9.22	22.10 ± 5.92 *	41.93 ± 9.19	35.33 ± 8.36 *	40.98 ± 4.49	27.94 ± 4.49 **
AP amplitude (mV)	66.20 ± 3.51	66.80 ± 2.68	62.50 ± 3.39	60.86 ± 3.77	62.04 ± 2.71	62.29 ± 2.55
AP threshold (mV)	−45.56 ± 1.29	−47.92 ± 1.53 *	−45.56 ± 1.40	−46.48 ± 1.66 *	−46.41 ± 1.08	−48.14 ± 1.26 **
Maximal rising slope (mV/ms)	233.80 ± 24.50	237.20 ± 20.11	200.58 ± 26.61	192.33 ± 25.17	203.06 ± 20.92	202.79 ± 17.06
1/2 width of AP (ms)	0.78 ± 0.09	0.83 ± 0.12	1.04 ± 0.13	1.07 ± 0.12	0.92 ± 0.09	0.93 ± 0.08
Afterhyperpolarization (AHP; mV)	13.50 ± 2.21	12.50 ± 2.20 *	11.59 ± 1.20	10.28 ± 1.23 *	14.95 ± 1.76	13.59 ± 1.91 **
R_N_ (MΩ)	310.17 ± 49.18	376.33 ± 54.10 **	413.00 ± 94.90	570.00 ± 147.11 *	421.57 ± 42.27	535.00 ± 62.24 *

## Data Availability

Not applicable.
